# Superabsorbent Core/Shell Composite Materials: A Review on Synthesis, Design and Applications

**DOI:** 10.3390/polym17111461

**Published:** 2025-05-24

**Authors:** Maria Pastrafidou, Evangelia C. Vouvoudi, Vassilios Binas, Ioannis A. Kartsonakis

**Affiliations:** 1 Laboratory of Physical Chemistry, School of Chemistry, Aristotle University of Thessaloniki, GR-54124 Thessaloniki, Greece; pastrafm@chem.auth.gr (M.P.); vbinas@chem.auth.gr (V.B.); 2 Laboratory of Polymers and Colours Chemisty and Technology, School of Chemistry, Aristotle University of Thessaloniki, GR-54124 Thessaloniki, Greece; evouvoud@chem.auth.gr

**Keywords:** nanoparticles, core/shell polymers, wastewater treatment, textile industry, water absorption, environmental impact

## Abstract

Superabsorbent core/shell composite materials are a type of advanced materials presenting enhanced water absorption and retention capabilities. The central core material can swell and absorb water covered by a shell that serves a specific function. The composition and functionality of each layer can be tailored to improve the material’s performance. The core is typically fabricated from superabsorbent polymers such as sodium polyacrylate, poly(acrylic acid) or other hydrophilic materials. The shell can be either inorganic polymers or organic polymers such as poly(methyl methacrylate), biodegradable polymers, polysaccharides or other functionalized materials in order to enhance biodegradability, mechanical strength or responsiveness to stimuli (e.g., temperature, pH). These materials present enormous potential to address issues for versatile applications in various fields, including biomedical applications, hygiene products and agriculture, due to their tailored structure. The common synthesis techniques for these advanced materials are emulsion polymerization, in situ polymerization, suspension polymerization with respect to the core material, layer-by-layer assembly and the sol–gel technique with respect to the shell formation. The techniques that are usually utilized for the characterization of the aforementioned materials and the validation of their functionalities are based on thermal analysis, morphology studies and swelling behavior and water retention and release mechanical properties, respectively. This review offers an in-depth examination of recent advancements in synthesis methods, structural engineering approaches and emerging applications of superabsorbent core/shell composites, highlighting the critical importance of material design in boosting their performance and broadening their practical use. Finally, special attention is devoted to the future perspectives of superabsorbent core/shell composites, exploring potential innovations in material design and multifunctionality. Emerging trends such as stimuli-responsive behavior, sustainability and scalability are discussed as key factors for next-generation applications. The review also outlines challenges and opportunities that could guide future research and industrial implementation.

## 1. Introduction

Polymers found in nature may be more or less hydrophilic depending on the chemical structure and are biodegradable over time. Synthetic polymers are used in the vast majority of applications nowadays, providing all the characteristics and properties required through their macromolecular construction. Furthermore, blended polymers, copolymers or grafted polymers have enhanced chemistry and capabilities, providing various profiles in terms of physicochemical properties. Therefore, hybrid and composite materials provide mechanical endurance when polymers alone do not [1].

Advanced materials are novel materials researchers manufacture by combining traditional ingredients in order to inherit special functions and properties. This is when the micro- or nanoscale of the structures is considered essential and attention has been garnered at the molecular and macromolecular level. Novel classes include gels and hydrogels, where cross-linking or polycondensation is evident but to an extent allowing hydrophilic groups to act. In that framework, superabsorbent materials, known for their remarkable capacity to absorb and retain large quantities of water or biological fluids relative to their weight, have attracted widespread interest across numerous sectors, including agriculture [2,3,4], hygiene and biomedical engineering [5,6,7,8], as well as environmental protection [9,10,11,12]. Within this broad category, superabsorbent core/shell composite materials have emerged as a particularly promising group of advanced functional systems [13]. By combining the distinct properties of core and shell components, these composites exhibit improved absorption efficiency, mechanical resilience, stimulus responsiveness and controlled release behavior. The core/shell architecture offers a flexible framework for fine-tuning material properties through the strategic selection of core materials, shell matrices and surface modifications, thereby optimizing their performance for targeted applications [14].

**Figure 1:**
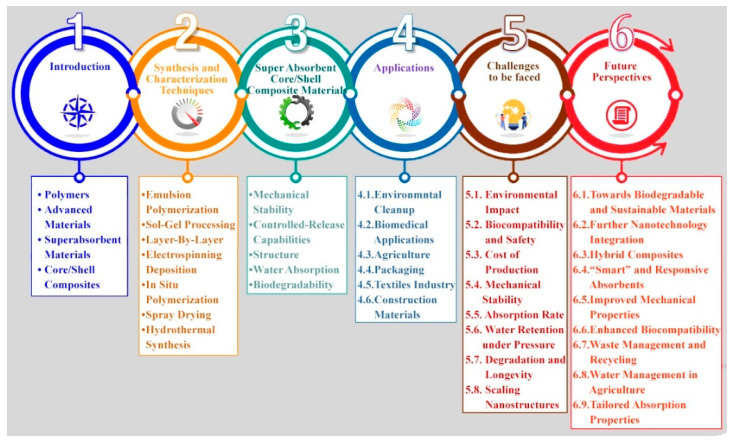
The framework of the review study.

The fabrication and design of superabsorbent core/shell composites employ a variety of synthesis approaches, such as in situ polymerization, layer-by-layer assembly and sol-gel processes, each contributing unique advantages in terms of structural control, scalability and durability. Ongoing innovations have significantly broadened the application scope of these materials, introducing smart functionalities like pH-responsiveness, thermal sensitivity and biodegradability. This review study provides a comprehensive overview of the recent progress in synthesis techniques, structural engineering strategies and emerging application areas for superabsorbent core/shell composites, emphasizing the pivotal role of material design in enhancing their performance and expanding their real-world utility (Figure 1). Finally, the review places particular emphasis on the future outlook of superabsorbent core/shell composites, focusing on prospective advancements in material design and multifunctional capabilities. Additionally, the study identifies key challenges and opportunities that may shape future research efforts and practical deployment. Their promising qualities prompt us to study and review them in depth and we present the findings of this search in the following sections [15].

## 2. Synthesis and Characterization Techniques of Core/Shell Superabsorbent Composite Materials

In general, the synthesis of core/shell superabsorbent composite materials (SACMs) includes techniques like emulsion polymerization, sol–gel processing and layer-by-layer and electrospinning deposition. Emulsion polymerization is the most common technique in order to synthesize core/shell structures, taking into account that different monomers or polymers are utilized for the shell and core components. This technique can be used for precise control over the size of the particles, their morphology and composition. The monomers in the core and the shell can be different, allowing the material to have unique functional properties, since it is tailored. Emulsion polymerization is suitable for many applications, like drug delivery. The risk of runaway reactions is minimized, and this can lead to good thermal control. The production of higher molecular weights can be possible due to the stable and fast polymerization rates. Unfortunately, some residues might be left behind, reducing the product’s final purity and performance [16].

On the other hand, sol–gel processing is effective for the production of inorganic core/shell composites, mainly those required for catalysis and water purification. This technique is based on the transition of a system from liquid (sol) into a solid state (gel). This also allows the precise incorporation of inorganic materials into the core. The composition and porosity as well as the surface properties of the shell can be controlled. Using this technique, a low-temperature synthesis is expected, that can be beneficial for incorporating temperature-sensitive components. Precise composition and structure can be achieved, since the mixing of the precursors in the sol stage leads to creating pure materials. However, it is a technique that is very time-consuming and the precursors can be very expensive. Moreover, the mechanical strength is limited and very sensitive to moisture [17].

Furthermore, layer-by-layer and electrospinning deposition are techniques that are rather applied for the precise control of properties and thickness of the shell layer. These methods are used in order to achieve better structural characteristics and functional performance of the shell layer. So, parameters like shell thickness, porosity and mechanical strength can be controlled. The layer-by-layer approach enables the sequential deposition of modified materials, so they can form uniform multilayered structures. As far as layer-by-layer assembly is concerned, a wide range of materials can be used, including polymers, in mild conditions. So, it is ideal for sensitive materials. Despite that, it is a very time-consuming process as well, since the deposition of individual layers can take time. Also, it is a technique that cannot be used in industrial volumes. For the electrospinning deposition, the use of high-voltage power supplies must be highlighted [18].

In situ polymerization is another way of producing these composite materials, with tailored shell thickness and precise properties, during which a pre-synthesized core material is suspended in a solution of shell monomers that polymerize on the core. A core material that is usually pre-synthesized is suspended in a monomer solution, with initiators, stabilizers or surfactants. Once the polymerization begins, the monomers polymerize onto the surface directly, leading to the formation of the shell layer. Strong interfacial bonding between the core and the shell layer can be accomplished by this technique. In-situ polymerization is a very adaptable method among advanced materials; however, it can be sensitive to factors like pH and temperature, limiting the choices of the materials. Moreover, side products might be formed so the reaction has to be controlled [19].

Furthermore, it may be remarked that spray drying is a fast and scalable technique that can produce a range of particle sizes with varied shell thicknesses. It is a rapid and cost-effective technique that involves the atomization of a liquid feed into a hot drying chamber. Then the solvent evaporates and this leads to the formation of dry particles. Also, it is a technique that can be used in fast and large-scale production. As before, it is a technique that allows controlled particle size and morphology [20,21].

Similarly, seeded dispersion polymerization is another method of producing SACM, with precise control over particle size, structure and shell composition. In this method, pre-formed seed particles that often make up the core are dispersed in a continuous phase, with monomer stabilizers and initiators, like before. The polymerization that occurs in the presence of these seed particles can lead to the controlled deposition of a polymer shell onto the core surface. This approach is better for materials with functionalized surfaces or multilayered shells [22].

Lastly, hydrothermal synthesis is a technique that requires heating the core materials in a high-pressure, aqueous environment that includes the formation of a shell layer around the core. This technique produces SACMs with high crystallinity and stability, ideal for high-durability purposes. The method involves subjecting the core materials to higher temperatures and pressures in a sealed aqueous environment, with the presence of metals salts. In these conditions, a shell layer is gradually formed around the core through crystal growth processes. This leads to the formation of uniform and thermally stable shell structures. Materials used under harsh conditions can benefit from this method. So, this method tends to promote slow and controlled crystal growth with precise control over particle shape and size. Nevertheless, this process requires high pressure and temperature and it is also expensive [23,24].

The produced core/shell SACMs are characterized in order for specific applications to be optimized by adjusting factors such as absorption rate, porosity and mechanical strength. The techniques that are used for the characterization of core/shell SACMs include scanning electron microscopy (SEM) to investigate the surface morphology and core/shell structure, thermogravimetric analysis (TGA) to estimate the thermal behavior, Fourier transform infrared spectroscopy (FT-IR) to determine the composition and chemical bonding of the composite, energy-dispersing X-ray analysis (EDS) for the element determination of the shell structure and swelling and absorption capacity testing in order to estimate these properties of the produced materials. SEM provides images that enable detailed examination of the surface morphology of the material, allowing access to information like particle shape, surface texture or even the quality of the shell coating. The uniformity and continuity or the core/shell architecture can also be confirmed by using SEM. Moreover, features like shell thickness, particle aggregation and surface roughness can be examined as well [25]. TGA can provide information about the thermal stability of the material and the decomposition temperature, which are very important. The shell thickness can also be determined by TGA by the estimated weight loss. This information is useful, since SACMs are often intended for high-temperature and harsh environmental applications. With FT-IR, functional groups and molecular interactions can be identified. Successful incorporation of functional groups into the core can also be detected, as well as some residual monomers [26]. As far as EDS is concerned, it can provide insight into the uniformity and distribution of the shell components. The successful deposition of the functional or inorganic shell layers can also be detected. This can be helpful to observe any impurities. Some other techniques that are also used are transmission electron microscopy (TEM) for detailed images at the nanometer scale, allowing precise measurement of the shell thickness, differential scanning calorimetry (DSC) for the observation of the thermal transitions of the material like melting, dynamic light scattering (DLS) to measure particle size distribution and zeta potential in suspension, Brunauer–Emmett–Teller (BET) surface area analysis to measure the specific surface area and pore size of the material and, finally, nuclear magnetic resonance (NMR) spectroscopy to analyze the molecular structure [27]. Like SEM, TEM can also provide high-resolution imaging at the nanometer scale. It is a technique that helps analyze the internal structure of the core/shell SACM, and in this way the shell thickness can be measured. Unlike SEM that can detect only the surface, TEM can give us information about the internal morphology of the shell. Multilayered or gradient shells can be confirmed as well [28]. Moreover, DSC provides insight into the thermal stability and phase changes of both core and shell components. This is useful in order to understand the material’s performance under different conditions [29]. DLS is a technique that can give us data about the hydrodynamic diameter of particles. The zeta potential can also be measured, that can help us predict the material’s behavior in suspension by giving us details about the surface change and electrostatic stability of the particles. These are properties that are needed in systems where dispersion quality affects the performance of the material [30]. The pore size can be measured by BET surface area analysis, as mentioned before. A larger surface area indicates greater potential for adsorption. The porosity of the shell layer can also be determined and can influence diffusion rates or the encapsulation efficiency.

## 3. Superabsorbent Core/Shell Composite Materials

SACMs present a variety of properties, such as elevated absorption, mechanical stability and controlled release capabilities, that make them a valuable solution in many fields and applications [31]. They are highly considered for their unique structure and their excellent properties that boost their performance. Specific functionalities like water absorption, mechanical strength and biodegradability can be improved due to the core/shell architecture that allows the coexistence of different materials [32].

The core part typically ensures chemistry and absorption capability, while the shell is constructed to enhance stability, control swelling behavior and introduce specific surface properties. This modification empowers the customization of these materials for diverse applications [33]. The core/shell design often leads to boosting resilience against leaching and material breakdown, ensuring prolonged functionality, even under harsh conditions [34,35]. Their ability to efficiently manage moisture retention and release and mechanical durability makes core/shell SACM a top-tier option for dealing with the challenges faced in many industrial and consumer applications [32].

The core/shell design allows SACMs to be adjusted for specific absorption targets, like selective uptake of water over oils, making them suitable for specialized tasks [36]. Moreover, responsive absorption based on environmental triggers can be possible, since materials can be synthesized to respond in changes of the pH or temperature [32]. This can adjust their absorption release rates accordingly, functionality beneficial for fields like agriculture. Furthermore, it should be mentioned that SACMs can also be biocompatible, thus they are safe for applications in healthcare products like wound dressings. The core/shell structure enhances the material’s resilience to breakdown, ensuring durability and cost savings in applications that require longevity [33,37].

The structure of such materials can be designed with varying densities, particle sizes and mechanical strengths to respond to the demand of applications that require flexibility [34]. Additionally, by integrating sustainable and degradable components into the core or the shell, SACMs can become eco-friendly and minimize environmental footprint [38]. Finally, SACMs can deliver nutrients, fertilizers or other chemical substances in a controlled way, allowing a slow and managed release that minimizes waste while maximizing effectiveness [39].

## 4. Applications

**Figure 2:**
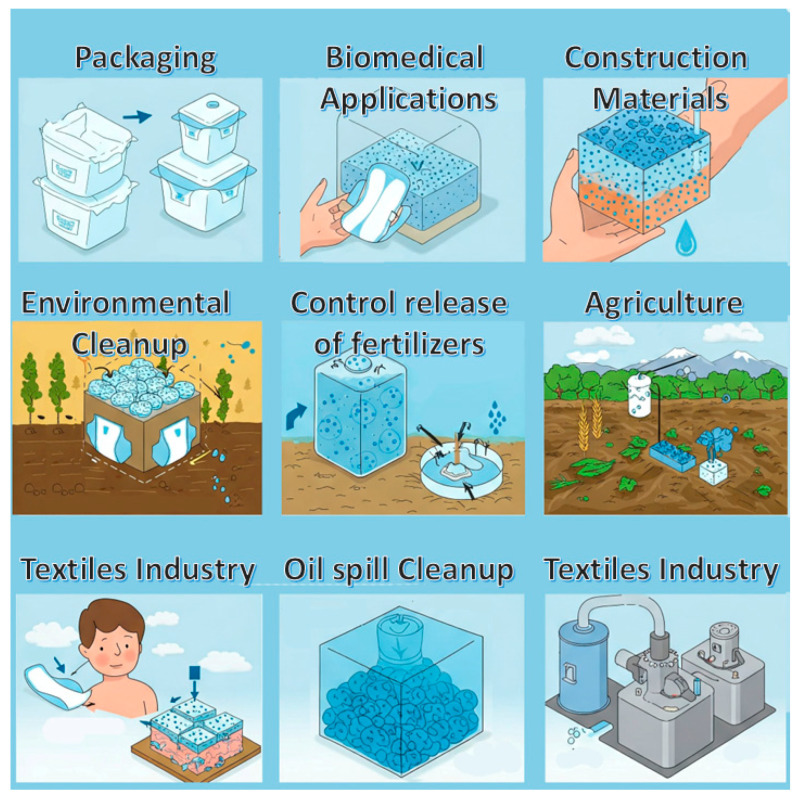
Summarized illustration of SACM applications.

The most important among the various properties of SACMs is their remarkable ability to absorb and retain great amounts of liquid with respect to their own mass. These materials are usually appreciated in applications where controlled release, moisture management or high absorption is very important. Their tunability and versatility make them ideal candidates for innovations in several fields such as environmental cleanup, biomedical applications and healthcare, agriculture, packaging, construction materials or textiles (Figure 2).

### 4.1. Environmental Cleanup

**Figure 3:**
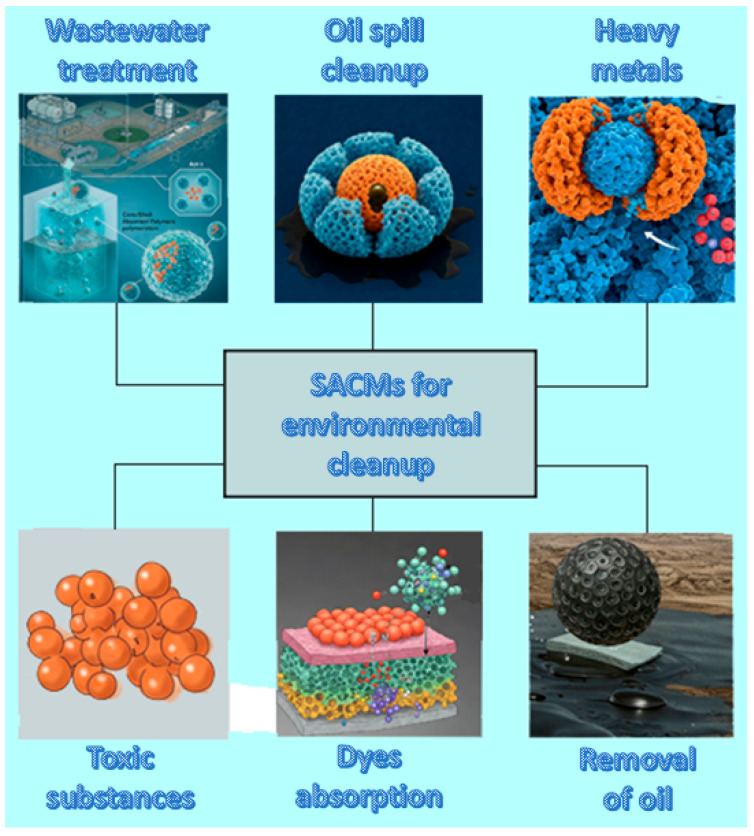
Summarized illustration of functions of SACM in environmental sector.

Taking into account that core/shell SACMs are able to absorb large quantities of pollutants dispersed in water, they are widely explored in wastewater treatment or oil-spill cleanup. Pollutants found in nature may be heavy metals, drug residues, toxic substances, dyestuffs or mordants and agents from industrial wastewater (Figure 3). Their functional surface groups as well as their high porosity make them able to trap pollutants efficiently. Furthermore, these materials can be employed for environmental cleanup through the absorption and removal of oil from the surface of water.

Nowadays, wastewater is burdened with drugs and their hydrolysis products. Solid-phase extraction used in wastewater-based epidemiology is time-consuming and labor-intensive just to extract some traces of the compounds from wastewater. So, a new polydopamine (PDA)-functionalized core/shell magnetic mesoporous silica (Fe_3_O_4_@*n*SiO_2_@*m*SiO_2_@PDA) nanocomposite was synthesized that not only could easily adsorb these compounds but could also be used at least 10 times without losing its adsorption efficiencies. This core/shell magnetic mesoporous silica nanocomposite was used for the extraction of five abused amphetamine-type stimulants from wastewater, such as methamphetamine and its metabolites amphetamine, ecstasy and methcathinone. Finally, the results showed that this material demonstrates strong potential for application in the future. The synthesis strategy includes, first, a silica shell coating on the surface of an Fe_3_O_4_ magnetic core via the condensation of tetraethoxysilane (TEOS) at room temperature. Successively, an outer layer of silica was synthesized by the hydrolysis of TEOS and mesoporosity was formed by removing porogen. Finally, the functional group of PDA was wrapped on the surface of Fe_3_O_4_@*n*SiO_2_@*m*SiO_2_ by self-condensation of dopamine. The obtained Fe_3_O_4_@*n*SiO_2_@*m*SiO_2_@PDA nanoparticles could be applied to capture amphetamine-type stimulants from wastewater samples [9].

On the other hand, oily wastewater has also caused issues in the environment. The literature refers to many materials that have been made in order to overcome this problem. One of those materials is nanoporous carbon aerogel nanoparticles (NPCA@C) that have been synthesized though a core/shell growth method. Nanoporous carbon aerogel serves as an excellent model for growing NPCA/C NPs and has an exceptional capability to remove formed *δ*-Al_2_O_3_ NPs using only heat. Aerogels (organic, inorganic or hybrid) are a unique and novel class of materials due to their low density, high porosity, large surface area and interconnected pore structure. Their properties are mostly attributed to their final condensation [10]. They were found to be an exceptional sorbent for the separation of both surfactant-free and surfactant-stabilized water-in-oil emulsions by gravity. This aerogel demonstrated outstanding separation performance even after 40 recycling cycles, showing no noticeable decline in its efficiency [11].

For the adsorption of heavy metals, such as Hg(II), Pb(II) and Cd(II) from artificially created high-salinity wastewater from coal processing, amine-functionalized Fe_3_O_4_ magnetic nanoparticles altered by an organodisulfide polymer, based on poly(trimethylene terephthalate), were designed. The characterization of these compounds was carried out by SEM, TEM, FTIR, BET, vibrating sample magnetometry (VSM), TGA and lastly XRD. Factors like pH, initial ion concentration, adsorption capacity and adsorption time have been taken into consideration. In wastewater with elevated salt levels and the presence of inorganic compounds, the adsorption efficiency of these functionalized magnetic NPs on polymer for heavy metals is outstanding [12].

Taking into account dye adsorption from wastewater, a core/shell bead adsorbent has been created by poly(acrylic acid) (PAA) microgels and polyethersulfone (PES). To form these core/shell beads by the phase inversion technique, PAA microgels have to be synthesized by distillation precipitation polymerization followed by wrapping with PES films. Using SEM and EDX it was confirmed that those core/shell compounds have PAA microgels in the core and PES on the shell. These beads are recyclable and can adsorb substances like methylene blue, methyl violet, rhodamine B, amaranth red and methyl orange. So, as a result, these beads showed potential for dye removal [40].

Xanthate is the most commonly utilized flotation reagent in mineral processing. It causes damage to the environment and it is a great problem in the construction of “green” mines due to its flotation in wastewater. In order to remove xanthate from flotation wastewater, novel copper-based core/shell adsorbents with coarse particle size have been created by the hydrothermal crystallization-surface replacement method for xanthate removal. Then, these compounds were characterized by SEM, TEM, XRD, FTIR, X-ray photoelectron spectroscopy (XPS) and N_2_ adsorption–desorption. CuO-based adsorbent has a higher adsorption capacity and the adsorption rate of potassium ethyl xanthate is faster than with Cu_4_(NO_3_)_2_(OH)_6_-based adsorbents. Due to electrostatic repulsion, the adsorption capacity is reduced by increasing solution pH and by adjusting the dosage removal of 99% potassium ethyl xanthate can be achieved. So, these copper-based core/shell adsorbents demonstrate great potential for application in the treatment of flotation wastewater [41].

Phthalic esters are common plasticizers used as polymer additives. Due to their great volatility, they are released by plastic surfaces, giving the characteristic odor of “new plastic”. They have estrogen-like effects that interfere with the endocrine system and potentially they can cause health problems. So, they are undesirable but they are difficult to detect. A novel core/shell magnetic mesoporous surface molecularly imprinted polymer (Fe_3_O4@SiO_2_@*m*SiO_2_-MIPs) was made, that had specific adsorption and rapid adsorption rates for phthalates. A rapid, efficient and sensitive method combining matrix-dispersive magnetic solid-phase extraction with gas chromatography–mass spectrometry (GC/MS) was developed using this composite as a magnetic solid-phase extraction material, specifically for analyzing the esters in different liquid samples. The adsorption isotherm and kinetics of this material indicated that it demonstrated fast adsorption rates, elevated adsorption capacities and finally high selectivity for phthalates. This suggests that this new method was perfect for their determination in water, alcohol, refreshments etc., due to its excellent sensitivity, superior efficiency and versatile sample application [42]. This application of SACM may be beneficial for both environmental and human health aspects.

### 4.2. Biomedical Applications

**Figure 4:**
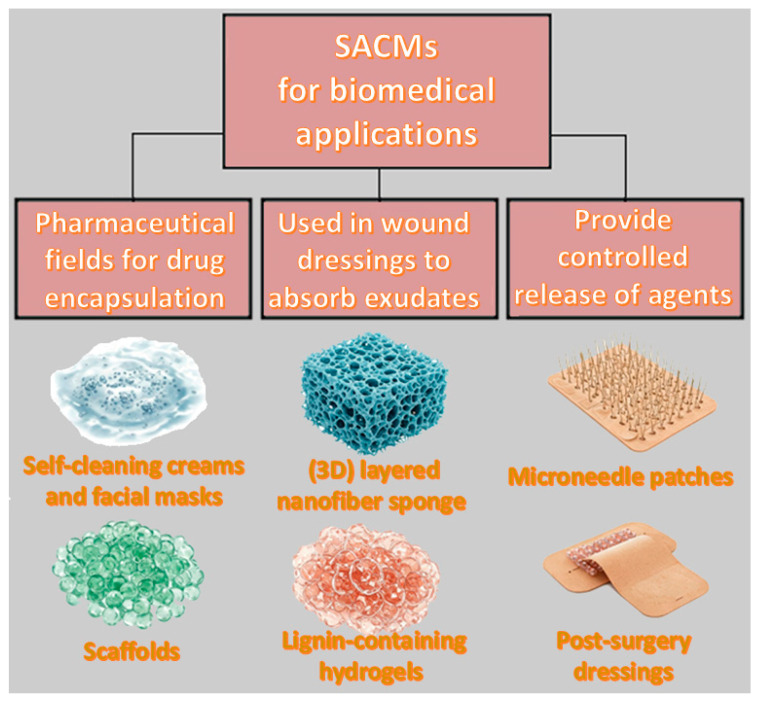
Fundamental applications for SACM in healthcare sector.

Drugs benefit from moisture control as well as the slow release properties of core/shell SACMs, making them suitable candidates for wound dressings and drug delivery systems (Figure 4). The core/shell composites can be used in the pharmaceutical field for drug encapsulation and, subsequently, their controlled release over time. The shell imparts protection while the core is able to store active agents, allowing site-specific and controlled release triggered by external temperature or pH stimuli. Furthermore, superabsorbent materials are integrated into wound dressings in order to absorb exudates, while keeping a moist environment that encourages faster healing.

Microneedle (MN) patches could be a hopeful treatment for diabetic foot ulcers, which concern many people. A thermo-responsive microneedle patch with high biocompatibility and without extra equipment is proposed. The thermo-responsive microneedle patch was prepared by combining the core/shell MN structure with a nanofiber membrane and it consisted of a bilayer of microneedles composed of a sodium alginate (SA)-*g*-poly(*N*-isopropylacrylamide) layer (SA-*g*-PNIPAM) loaded with sucrose octasulfate sodium salt and a hyaluronic acid layer and a polycaprolactone/chitosan nanofiber membrane loaded with tetracycline hydrochloride and sucrose octasulfate sodium salt. The MN patch is thermally responsive and can self-operate the transportation of the drugs without additional devices, speeding up drug release while improving the application of the drug. The salts boost wound healing by blocking bacterial growth and promoting vascular regeneration and epithelial formation. The thermo-responsive MN patch had good biocompatibility and great healing effects in vivo. The results indicated that the drug release was fast, as the drug release rate was more than 80% with the antibacterial rate reaching up to 800% [5].

BioBran, developed and marketed as a hydrophilic, non-toxic and safe bioactive agent/supplement, sounds interesting regarding the immune system. BioBran may be used as a bioactive substance that could optimize wound healing, since it affects the properties of nanofibrous scaffolds created through coaxial electrospinning. The scaffolds were composed of a poly(*ε*-caprolactone) (PCL) shell and the core consisted of various concentrations of BioBran blended with poly(vinyl alcohol) (PVA). The scaffolds loaded with BioBran demonstrated a more condensed and smooth morphology in relation to the one with no BioBran. BioBran also positively affected the physical interaction and crystallinity of the polymers in the scaffolds in a concentration-based approach. It helped with their tensile strength, elongation at break, thermal stability and hydrophilicity. The release rate for BioBran had a biphasic pattern and decreased with the increase in its concentration, ensuring managed and maintained delivery from the nanofiber scaffolds. All of that suggested that BioBran-loaded core/shell nanofiber scaffolds offer prospective applications in wound healing as an optimal multifunctional wound dressing [6].

Similarly, the control of bleeding is very crucial. Traditional wound dressings cannot control bleeding. So, a three-dimensional (3D) layered nanofiber sponge was created by expanding two-dimensional (2D) nanofiber membranes into 3D structures. The interfacial interaction between the sponge and the blood cells in order to accelerate hemostasis was increased by the layered nanofiber structure. This 3D nanofiber sponge has properties beneficial to wound healing and also good elasticity, high permeability and a high fluid absorption capacity. Moreover, it is highly compressible and resilient, offering effective tamponade for deep wounds while establishing a robust 3D dynamic microenvironment that supports and regulates cellular behavior. The layered nanofiber sponge is composed of chitosan and PVA. The positive charge of amino groups in chitosan interacts synergistically with the negative charge of platelets, enhancing and accelerating the hemostatic process. The PVA is incorporated to enhance chain entanglement and increase the water solubility of electrospun chitosan by modifying the intermolecular forces. So, sponges with adjustable thickness and porosity can be produced with this method, so this porous nanofiber sponge has strong promise in the future for clinical applications and wound dressings due to its good wound-healing property [7].

Hydrogels are typically described as cross-linked, three-dimensional polymeric networks that can not only absorb but also retain a large amount of water. Lignin is a natural polysaccharide macromolecule with high aromatic content that is the most common sustainable energy source. The development of lignin-containing hydrogels seems a promising topic, and they can be used as absorbents in wound dressings and strain sensors. There are multiple covalent and non-covalent interactions between lignin and the polymeric matrix that equip hydrogels with many functionalities. Wound dressings exploit the antioxidant activity of lignin, and strain sensors benefit from the mechanical strength and flexibility of these hydrogels. Overall, lignin-containing hydrogels show great potential in future applications [8].

Most healthcare problems are related to the existence of pathogens like bacteria, viruses or fungi and superinfection or hospital cross-infections. This is a result of colonies expanding into big clusters and forming spatial networks within the polymer matrix or biofilm that is composed of exopolysaccharides generated from the bacterial cells directly and they tend to persist on materials like plastic, metal or even cotton. In order to clean surfaces that could be exposed to bacteria and complex biofilms, with accompanying absorbed antibiotic compounds, a self-cleaning material in the form of a suspension was created. It included beeswax, with cetyltrimethylammonium bromide (CTAB)-assisted synthesis of cubic-shaped Cu_2_O nanostructures. This material has a cubic morphology and a beeswax shell and a well-defined Cu_2_O core. The Cu_2_O/beeswax/CTAB materials show powerful antimicrobial properties against prevalent bacterial and fungal strains, such as *E. coli*, *S. aureus* and *C. albicans*, and one spray of these can kill bacteria in the biofilms. In conclusion, the self-cleaning and antibacterial properties can also be combined for more practical applications in the future [43].

Core/shell SACMs are explored for managing chronic conditions and in post-surgery dressings, where managing exudates is very important for recovery. They can be used in hospital settings to control liquid medical waste, such as bodily fluids or blood, among other medical applications. Taking into consideration that core/shell SACMs reveal enhanced liquid retention as well as improved absorption properties, these materials are widely used in adult incontinence products, sanitary napkins, diapers and other hygiene products.

Finally, regarding personal care and cosmetics, core/shell composites are able to hold water or hydrating agents and therefore can be used in gels, moisturizing creams and facial masks on a large scale. The material at a slow “home”-related pace releases moisture over time, resulting in longer-lasting hydration. In deodorants, these materials assist in moisture absorption, neutralizing odor while keeping skin dry.

### 4.3. Agriculture

**Figure 5:**
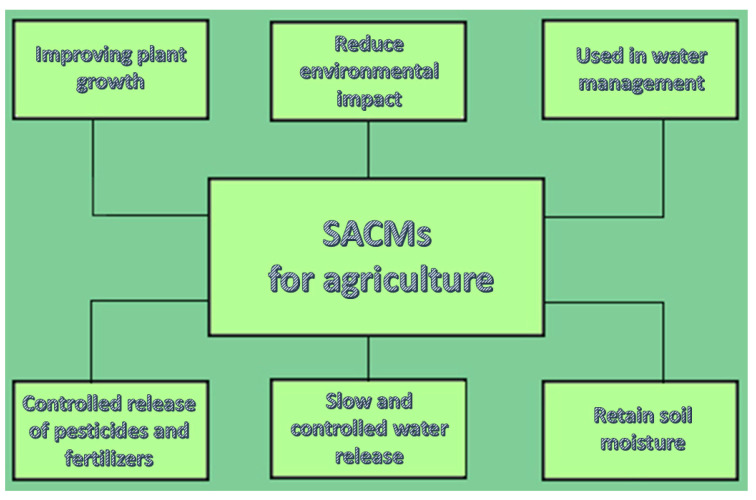
Division of agricultural applications of SACM.

A very common usage of core/shell SACM is in water management in order to retain soil moisture, specifically in arid regions. The core/shell structure enables slow and controlled water release, improving plant growth through the reduction of irrigation frequency. Moreover, a controlled release of pesticides and fertilizers can be applied because core/shell SACM can encapsulate pesticides or fertilizers, releasing them at a slow pace taking into account the soil moisture conditions, thereby improving the efficiency of the input and lessening environmental contamination [2]. Figure 5 shows those applications.

The Cd^2+^ extraction from soil is very important as it enters the food chain and it is a serious threat to human health. A dual-functional core/shell sphere, PPC/PC-Fe, was created for efficient soil Cd removal. The core (PC-Fe) was composed of a poly(acrylic acid)/carbonxymethyl chitosan (PAA/CMC) hydrogel with Fe_3_O_4_ NPs in order to adsorb adjacent activated Cd and the shell (PPC) was made up of encapsulated citric acid in a poly(lactic acid) (PLA) and poly(ethylene glycol) network, which imparts the ability to activate Cd. Following contact with water, soil Cd was activated due to the citric acid that was released because of the desolvation of the shell. Then, the PC-Fe absorbed water and then swelled and expanded in size, that helped in the decomposition of PLA in the shell. This initiated the automatic separation of the core from the shell, finally enabling the exposed PC-Fe core to rapidly adsorb Cd. The PC-Fe core can then be magnetically removed. The use of 2% PPC/Fe removed 19.5% Cd from soil in 10 days [3].

In order to improve the efficiency of fertilizers and minimize their negative impact on the environment, a new fertilizer with slow release of nitrogen and boron from urea and borax with water retention was synthesized. As the basis for copolymerization, wheat straw was used, on which acrylic acid monomer was grafted to form superabsorbent composite. The product had a core/shell structure, of which the core was urea in attapulgite and alginate matrix, and the shell was chemically modified wheat straw-*g*-poly(acrylic acid)/attapulgite (CMWS-*g*-PAA/APT) superabsorbent composite consisting of urea and borax. In tap water, the water absorbency of the superabsorbent synthesized under ideal conditions was 186 g/g. To conclude, the product with slow release and water retention capacity is cheap, non-toxic in soil and not harmful to the environment, so it shows great potential for applications in agriculture [4].

Novel single- and double-coated water retention urea fertilizers were made with polyacrylonitrile (PAN) alone and with PAN-based poly(acrylic acid) (PAAc) hydrogels. The core consists of urea granules coated with three different natural oils that form the first shell, soybean oil, oleic acid and linseed oil. The second shell consisted of PAN and PAN-based PAA hydrogels. The polymerization reaction was performed with the use of *γ*-ray irradiation, the doses of which contributed to the examination of the slow release property of the urea, and the results indicated that both polymers had controlled the release. An alternative method for controlling the urea spillage is through the application of superabsorbent hydrogels, that can absorb and conserve a high volume of water, even under pressure. So, covering the urea with an appropriate hydrogel presented a good water absorbency in soil and displayed a slow release profile of urea. The synthesized urea granule coatings with hydrogels showed no antagonistic effect on plant growth-promoting rhizobacteria, so they were safer for utilization in agricultural fields [44].

### 4.4. Packaging

Superabsorbent composites are used in packaging to preserve the freshness of food through the absorption of excess moisture. These materials can be used in packaging films in order to prevent further water condensation, that can lead to degradation and spoilage of food products. Food packaging is a significant part of the packaging industry in Western economies and the most significant contributor of single-use plastic waste as well. Apart from the obvious roles in security, transportation and aesthetics, a new generation of packaging materials may serve as multiple components of the food chain sector [45]. Environmentally friendly and “active” food packaging, with no petroleum-based polymers, is of great interest, even more so when it involves the use of sustainable biomass resources. Due to their sustainability, biodegradability and accessibility, cellulose nanofibers (CNFs) can be used in constructions like films, membranes, hydrogels, aerogels, foams and microcapsules. This enables the exploration of the connection between the framework and effectiveness in active food packaging. To form cohesive films on the core, CNFs are usually used, accommodating the controlled release of the core. A formulation that contains the core and the encapsulant is dispersed into an emulsion in a solvent of oil in water (o/w) or water in oil (w/o), resulting in the creation of a tight polymer layer around the core. So, an active film was made, that encapsulated oil core/CNF shell microcapsules that were active against E. coli and L. monocytogenes. The results indicate that various properties of the CNFs have been promoted in order to achieve “green”, renewable and recyclable food packaging [46].

In the course of storage and transport of the demanding fish market chain, minimizing microbial contamination and controlling fluid exudation from the fish can significantly reduce spoilage and the degradation of fish fillets. Coaxial electrospinning films were loaded with natural plant preservatives, such as laurel essential oil and clove essential oil, and combined with nanoemulsion techniques and then hydrophilic preservation pads were made. The film fibers exhibit a clear morphology, free of beads or defects, with fiber diameters ranging between 230 and 260 nm and having a distinct core/shell structure, great thermal stability and excellent antibacterial and antioxidant properties. The core/shell structure gently controls the release of preservatives and greatly enhances the efficiency of utilization. The cooperative use of the two essential oils can minimize the amount while boosting their efficacy. The pads decreased the speed of the increase in key indicators of spoilage, such as total viable count, pH, thiobarbituric acid reactive substances and total volatile base nitrogen, during the storage of the fish fillets. The pads also delayed the reduction in water-holding capacity, the degradation of textural qualities and the adverse changes in the microstructure of the fish muscle. Eventually, the pads delayed the damage to fish fillets and extended their shelf life from 5 to 9 days. The effective use of biological preservatives in this film can offer technical assistance for the advancement of food preservation materials [47].

### 4.5. Textile Industry

**Figure 6:**
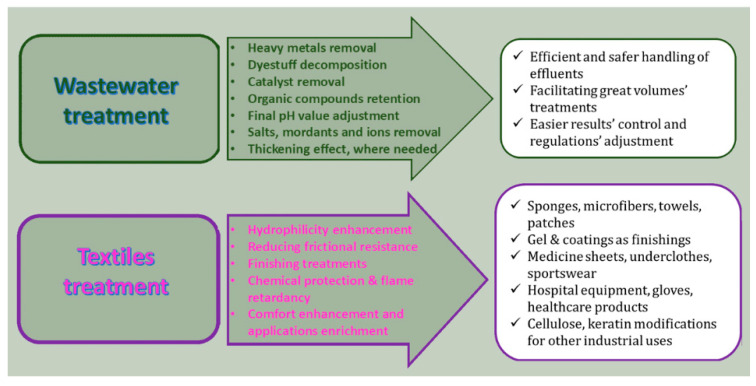
The contribution of SAPs in Textile Industry, for both environmental protection and advanced final products.

Two aspects of the textile industry incorporate the application of SACM: the production of moisture-control fabrics and the post-treatment of dyeing wastes (Figure 6). Particles may be adhered to textiles and fabrics designed for temperature and moisture-wicking control. Medical and sportswear textiles usually incorporate these materials for functionality and comfort. In hospital settings, SACMs serve in liquid absorption, improving usability and comfort.

Wastewater released by textile industries globally is generally burdened with various chemicals, such as organic compounds, ions and heavy metals, and this is hard to change. Advancements in post-treatments have been made regarding the management of dyes in wastewater and effluents, for protecting groundwater and environmental resources, in accordance with national regulations around the world. Tadayoni et al. proposed, for the removal of organic dyes from textile effluents, covalent triazine polymers (CTPs) with unrivaled capabilities. A CTP shell was made through condensation of melamine and cyanuric chloride on silica-coated functionalized magnetic core nanoparticles and then modified with amine functional groups. In this way, a flower-like magnetic core/shell covalent triazine polymer (MNP@*m*-CTP) with dispersed magnetic particles as “pistils” and a suitably thick the porous polymer shell as “petals” was prepared. The structure was confirmed by FT-IR, SEM, TEM, TGA, XRD and VSM. Then, MNP@*m*-CTP was used as an adsorbent in Direct Scarlet 4S (C.I.Direct Red 23, MW = 769) dye removal and the adsorbent efficiency was high, considering the influence of the main parameters. Ninety-five percent of the dye was cleared from the water within 240 min, with a max adsorption capacity of 215.28 mg/g [48].

### 4.6. Construction Materials

In construction, superabsorbent materials are being explored to enhance the performance and durability of concrete, helping in “self-healing” concrete through moisture absorption and preventing cracking. Core/shell SACMs can also be used as effective barriers to control moisture and prevent water infiltration, that can lead to material degradation.

In order to develop a self-healing cementitious material, a “green” capsule was created by combining sludge and calcium hydroxide and then microcrystalline cellulose and sodium polyacrylate were added to the core mixture to increase the healing efficiency. The capsules were almost spherical. The shell demonstrates a glass-like material, so the core is fully covered, dense and smooth. Results show that the plain capsule could heal cracks up to 400 μm, and the closure width can increase to 500 μm and 800 μm in 3 days [49].

A two-bacteria-capsule system was created in order to boost the self-healing capacity of expanded polystyrene, that tends to float during mixing and vibration. Poly(ethylene glycol) was used to encapsulate the core and a mixture of sulfoaluminate cement and epoxy resin was used to create the shell. Two bacteria capsules were created. The A capsule consisted of aerobic bacteria, superabsorbent polymers and expanded polystyrene and spontaneously rose during the composite preparation process while the N caspule consisted of anaerobic bacteria and SAP and, because of the extrusion effect, was distributed in the middle and bottom regions. Both were prepared through granulation and the process was carried out in the presence of glycerol. The findings revealed that the capsules fractured in unison with the composite material, while the coating effectively inhibited the premature release of the “self-healing” agents. Cracks measuring 50–600 μm in width were sealed up to 90% when double capsules were incorporated into cementitious composites [50].

## 5. Challenges to Be Faced

**Figure 7:**
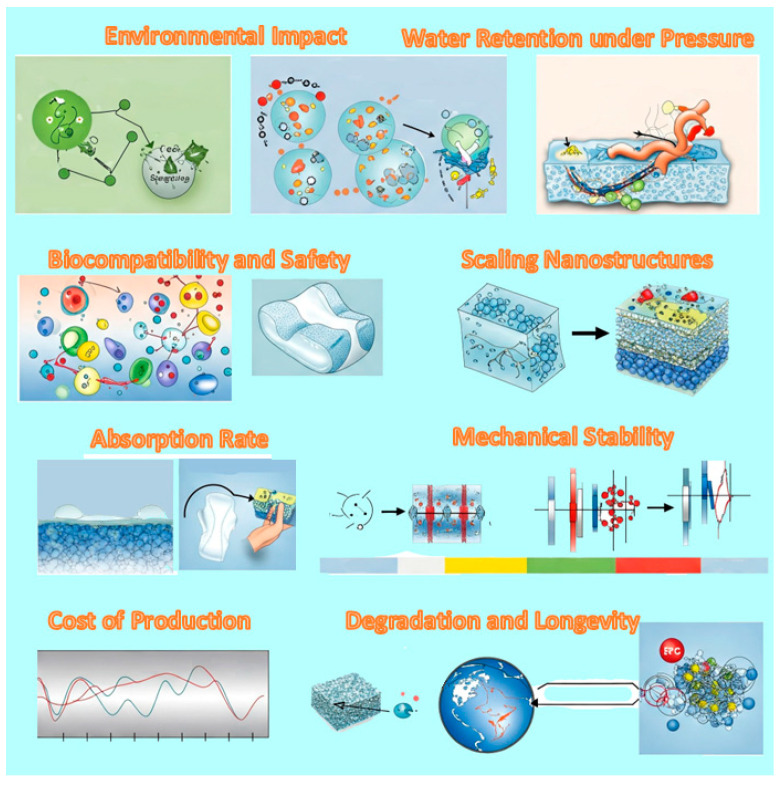
The classification of SACM challenges that need to be faced.

Core/shell SACMs are designed for enhanced water absorption and retention. While they already offer valuable possibilities, there are several challenges, as seen in Figure 7, that can affect their manufacturing, performance or environmental impact.

### 5.1. Environmental Impact

Core/shell SACMs face significant environmental challenges, including non-biodegradability, which leads to long-term pollution and the potential leaching of harmful chemical additives that can further contaminate soil and water. The degradation of these materials can result in microplastic formation, posing risks to wildlife and human health [39].

Core/shell SACMs are non-biodegradable by themselves, since they are based on synthetic macromolecular structures. The ones that are used in agriculture or constructive materials are designed to be durable and also long-lasting. These materials are made from synthetic polymers like polyacrylate, polyethylene and others, that do not break down naturally [51]. As a result, it takes time for them to decompose after their use and can lead to long-term pollution. They accumulate in landfills and remain there for, as estimated, hundreds of years and contribute to land degradation that makes it harder to restore healthy soil naturally [39]. It is noted that polymers are not toxic or harmful by themselves, since their macromolecular structures are too big to enter the cells of living organisms and harm them. So, their retarded natural decomposition, and thus the accumulation in landfills, water and the environment, makes them undesirable. Humans are eager to “vanish” polymers as wastes, so they do not cover the Earth, and they decompose slowly. In this process, H_2_O and hydrophilicity play a key role.

The issue appears more in agricultural areas due to the extended use of SACMs to manage water retention. Moreover, these materials contribute to the carbon footprint of the disposal process, because many are incinerated and produce greenhouse gases [52]. SAPs can also contaminate water and soils, since they contain chemical additives, like surfactants, plasticizers and stabilizers that leach into the environment and can contaminate water supplies, affect soil quality and generally affect wildlife and plant life [53].

Over time, these materials degrade into microplastics (<5 mm) that contribute to global warming and environmental pollution or even affect terrestrial and aquatic ecosystems and lots of animals, including humans [54]. More specifically, when animals ingest these microplastics, they suffer from physical harm like digestive blockages, malnutrition and reduced ability to absorb nutrients. All of that can lead to injury or death. Microplastics can act like vectors for toxic chemicals, such as pesticides, heavy metals and pharmaceuticals, that later enter the food chain [55].

In some other cases, chemicals found in plastics (as additives) may leach out and affect the hormonal system of animals to cause reproductive problems and often marine species lose their ability to perform essential functions such as foraging and migrating. These biological disruptions can have cascading effects on entire food webs, impacting biodiversity and ecosystem health [56]. By eating meat from affected animals, humans can also be affected by microplastics and other chemicals, causing reproductive issues, endocrine disruption and other health problems [57]. All of these can contribute to the bioaccumulation of coexisting harmful substances that pose a risk to ecosystems, whether they are aquatic or terrestrial, and humans as well.

### 5.2. Biocompatibility and Safety

To continue the syllogism of SACMs affecting the environment, the last link of the chain is humans. Biocompatibility and safety, especially in biomedical applications (see Section 4.2), are also included in the challenges that core/shell SACMs have to face. Synthetic plastics, and the chemical additives that go into making these products, may cause adverse biological responses, such as inflammation or toxicity, in biological tissues. They can also leach dangerous chemicals into the body which raises the question of whether they are safe and effective [57,58].

When core/shell SACMs are implanted or used in contact with biological tissues, they can trigger immune responses [59]. This can potentially lead to inflammation at the site of the application while chronic inflammation can lead to further complications [60]. The immune system may form a protective fibrous capsule around the material, causing limitation to its functionality, and sometimes it may even be rejected. Materials that are designed to stay inside the body for a long period of time are the ones that raise the most concern. Moreover, synthetic polymers are not always recognized by the system and that can cause allergic reactions [61].

To enhance properties like flexibility, durability and resistance to degradation, chemical additives like plasticizers and stabilizers are added to SACMs. However, all these chemicals can leach out of the material and expose bodily fluids and over time can interfere in the hormonal system and cause concern regarding productiveness [59]. Compounds like these can also gather in tissues and generate a buildup of toxic substances in organs like the liver or kidneys. This may not show any symptoms in the beginning but it is assumed to lead to chronic toxicity and health issues. Unfortunately, leaching from SACMs can be unpredictable, since factors like temperature and pH can influence the rate and the extent of it [57,58].

Also, while SACMs may degrade eventually and be discarded from the body, their breakdown products can be harmful. For example, some polymers can degrade into acidic or basic components that are not good for the human body. This may not be the case at the beginning, but in due course their effect could lead to inflammation or even organ failure. Eventually, when the body becomes chronically exposed to potentially harmful chemicals, like heavy metals or persistent organic pollutants from SACMs, the risk of cancer increases, as well as that of cardiovascular issues or immune system suppression [61,62]. These heavy metals and some plasticizers can also interfere with cellular processes like gene expression, protein synthesis or even immune responses and normal tissue regeneration.

In order to achieve the properties that make SACMs attractive for biomedical applications, synthetic materials or additives are used, and they may not be entirely biocompatible. For example, by increasing the amount of SAP to improve fluid retention, the risk of potential leaching of harmful chemicals is increased as well. So, different medical applications may require specific design adjustments to optimize the balance between absorbency and safety [38].

### 5.3. Cost of Production

The complex synthesis of core/shell SACMs makes their production cost very high. Making particles out of composites may take some time, so the costs can go up because of that. Obtaining correct core/shell structures requires extra tools and technologies and hence incurs additional costs [63]. Thus, further research is needed to develop techniques and materials that are more abundant and more accessible.

To achieve the desirable properties of SACMs, high-quality raw materials are often used. These materials include special features, such as biocompatibility or hydrophilicity, that enhance their performance. Unfortunately, they are very expensive, and, in most cases, polymers used in SACMs require synthesis in controlled environments [49]. The procedure may be difficult and complex, and different solutions and solvents may be needed, all of which are expensive with high purity [64]. Moreover, additional chemical additives, stabilizers or cross-linking agents may be required, that adds further material costs. SACMs are often designed for specific uses, and customizing materials for different applications means more experiments with polymers and coatings that raise the cost of production.

Core/shell SACMs must comply with specific certifications and quality standards, that often require expensive testing, documentation and reporting. Most of the time, they must undergo rigorous testing and approval processes. These testing courses can be expensive as well, and the approvals can involve lengthy and costly procedures that require money as well. Also, the transition from small-scale to large-scale production that involves difficulties in maintaining the same material properties requires additional refinement in manufacturing processes and equipment [39]. So, larger facilities and machinery are needed, driving up the overall cost. The need to invest in new and better manufacturing equipment is expensive as well. On top of that, it requires skilled workers, with expertise in material science, a high-demand profession, with higher wages and labor costs.

Lastly, many of the raw materials and chemicals that are being used are not environmentally friendly. So, new waste reduction strategies or sustainable sourcing of materials must be implemented by the manufacturers. Replacing harmful chemicals with safer alternatives can significantly raise the cost of production [38].

### 5.4. Mechanical Stability

The fundamental property of any core/shell material is the transmission of stress and load to the inner particles. The well-known copolymer of polystyrene-co-polymethylmethacrylate particles functions accordingly, when the mechanical stress is transferred to the amorphous and higher-volume polystyrene while the glassy polymethylmethacrylate is protected from cracking at low stress. For absorbent materials, though, the significant water absorption capacity of core/shell composite materials represents a great challenge to their mechanical stability. The swelling causes the shell to experience volume and shape changes resulting in cracking, distortion and finally collapse of the shell. If core and shell are weakly adhered then, during the application of stress on the structure, it may produce a destroyed site which is known as debonding of core from shell. This difference in mechanical properties between core and shell may also disturb the stability. Therefore, the properties of composites’ durability depend primarily on improving their adhesion and mechanical behavior between the two phases [29].

When absorbing liquid, the shell is forced to stretch, and this creates internal pressure [65], while substantial stress is generated that leads to microfractures [66]. These microfractures in the shell seem small in the beginning, but in due time they become bigger and gradually create cracks. So, when high stress is needed, the integrity of the material can be compromised [49]. As soon as the shell fractures, the core might become unevenly distributed, due to the spillage of the absorbent core, and can no longer absorb liquid [49]. Cracks can also create leaks; therefore, in the case of liquids in cores they can seep out, and this makes the material not reliable.

Every time the core swells by absorbing liquid and then contracts upon drying, both core and shell stretch and shrink back [65]. If this happens many times, mechanical fatigue might be created, causing the degradation of the materials’ stability [49]. Also, when the core is wet, properties like structural rigidity can be lost, and softness can cause tearing or deformation. This deformation might be permanent, due to the repetitive stress cycles, resulting in a less effective fit and also reduced absorbency [49]. Cycles like these decrease the ability to bounce back and those materials are not suitable for repeated use, as they present some limits in their lifecycle.

Moreover, the stiff outer shell is not designed to impart flexibility in the material. Properties like bending, twisting or compression are needed in some applications, and without them, limitation to the material’s ability to adapt to energetic movements can occur. This can provoke cracks, so materials like them are not a great fit for applications where flexibility is required. Also, the firm shell might lower the absorption capacity, because the core is restricted and cannot expand to its fullest. The lack of elasticity and flexibility can limit the material’s durability and effectiveness over time.

The bond between the shell and the core can be affected when the core expands [40]. So, it is likely that they will start to detach from each other, especially in the layers that cannot withstand high stress. Swelling can diminish the adhesive bond between the layers and the structural integrity might lessen. Once the core and the shell separate, it is easy for the absorbent core to cluster or swift, and uneven absorption and retention are inevitable. This happens because the material can no longer hold its original amount of liquid, and it is less effective when high absorption is needed [36].

### 5.5. Absorption Rate

One big challenge with core/shell SACMs is getting them to soak up water at a steady rate. Differences in the core and shell can make water go in (sorption/swelling) and out (desorption/drying), which impacts how well they work overall. When the shell is too thick or made of certain materials, it can slow down how fast water moves through it [67]. This unevenness can make these materials less effective for applications that need to handle moisture quickly. Scientists need to do more work to make the structure and features of these materials better at absorbing water.

When SACMs are exposed to high external pressures, in applications like packaging or industrial use, the material is compressed. Due to external stress, the core can no longer expand effectively to take more liquid, causing a decrease in the absorption capacity. So, in applications in which varying external pressures occur, these materials are problematic. Moreover, when the material is exposed to high compression, the shell might lose its structure and, in the case of liquid, allow the liquid to seep through in an uncontrolled manner, even though outer shell is designed to protect the core and regulate liquid absorption.

The protective shell around the absorbent core can delay the absorption process, as the shell acts like a barrier that liquid must pierce to reach the absorbent core [68,69]. This can increase the absorption time, but this is not desired in applications where fast absorption is crucial. Also, the liquid must spread through the shell before it can be absorbed by the core. But when the shell is thick and dense, this procedure takes time and eventually slows the process down.

Shell thickness can impact absorption time in another manner too. An uneven absorption performance is often caused by the topological variation in thickness of the shell [12]. Unlike the thicker parts of the shell, the areas of the shell with a thin layer can absorb liquid quickly. Moreover, the importance of core/shell bonding is also a factor as this facilitates the passage of liquid from shell to core; the chemical affinity between the two provides secure bonding. In other words, if the coupling between them is too weak, liquid will not migrate evenly from the shell to the core and will eventually fail to penetrate some areas [70].

Under continuous exposure to moisture, heat and other environmental factors, the materials that are used for the composition of the core and the shell in SACMs can degrade, causing the degradation of the absorption ability [71]. Moreover, the core material might leach out in due time, and then the absorption properties will start to lessen, reducing the overall absorption capacity.

### 5.6. Water Retention Under Pressure

A factor that also challenges core/shell SACMs is the water retention capability when subjected to external load. Loads (e.g., stress, compression, shearing, bending, twisting) may lead to consolidation of internal structure or, more often, fracturing of the shell layer which would compromise moisture retention [49]. This lowers their potential in vital applications and stresses to retain moisture under cross-flow.

Water retention is reduced by compression that can be caused by pressure. If a small amount of external force is applied, or even in conditions of high pressure, the absorbed water might leak out of the core. As a result, the material’s performance in retaining liquids is degraded, and the water is released by the absorbent core, generating rapid leakage. In some cases, those materials cannot be used in long-term and high-retention applications, due to the inconsistent retention of the liquids that lowers their potential [65]. Also, this pressure can affect the overall ability of the material to swell completely and the core’s ability to reach its full absorbent volume. So, the maximum capacity to absorb and hold water is degraded, especially in cases where a force is constantly applied and the core cannot fully expand. As a result, portions of the liquid core content remain unsoaked, wasting potential absorbing power.

In situations of dynamic loading, like shifting or bending over time/temperature, fluid liquid can be leaked, because the material is no longer stable to hold the absorbed water. The material can also lose fluid by repetitive cycles of pressure moment, that can cause a reduction in the stability of retention, that is crucial for some applications. The internal structure can be compressed when long-lasting pressure is applied, reducing the available space for liquid to cover, resulting in a lower volume of water that can be retained, and this can often lead to lasting deformation of the material. The absorbing surface area can also be reduced in those cases [71].

Once the pressure is removed, the material needs time to re-expand to its original absorbent state, limiting its ability to reabsorb liquids quickly. Sometimes it is not possible for the material to return to its initial state, and if it does it is not certain whether the material will still be absorbent or not. Furthermore, in some multilayered structures, the pressure may affect some layers more. This means that uneven retention and fluid distribution are inevitable, reducing the overall efficiency of the product. These different compression levels across the layers can burden the bonds between them, and the layers can be divided, compromising the liquid retention. This is more common in thick multilayered absorbent products.

### 5.7. Degradation and Longevity

Core/shell SACMs may also face degradation and durability problems, such as exposure to UV rays and moisture, that can destroy the materials used [68]. Differential degradation rates of the core and shell can compromise performance and function. Improving the compatibility and environmental stress resistance of these composites could improve their lifespan in real applications.

Breakdown of the core material is possible over time and temperature. Every time a cycle of absorbing and releasing moisture is completed, the polymeric chain weakens and therefore it is easier to break down, especially in applications that require repeated use. The core materials degrade and the ability to attach and maintain water might be lost, lowering the overall absorbency of the materials. Also, degraded polymers may form clusters within the core, causing uneven distribution, and this creates areas that are less effective at absorption. Continuous exposure to pressure can cause cracks in the shell, as explained above, compromising its role eventually [71]. In environments with temperature or moisture variations, the shell can become brittle and prone to fracturing. As the shell weakens, the core is left unprotected and this may lead to the separation of the two, reducing the integrity of the overall structure. This separation could be similarly caused by prolonged moisture [72].

Environmental changes can also affect these materials, for example, extreme (in comparison to their working settings) temperatures can make the shell brittle or soft, accelerating degradation. High dampness may cause moisture absorption when the product is out of use, deteriorating both the core and shell, shortening their lifespan. UV is an important factor in premature aging and decreased durability as well. UV rays impose high bond energy that can directly break down the bonds in the macrochains in both the core and the shell, thus disrupting the polymeric skeleton and reducing its ability to swell and absorb moisture. Eventually, the core loses its flexibility and thus its water retention ability [72].

In high-friction or frequently used products, the shell may be destroyed, exposing the core material and causing leaks; this jeopardizes the wholeness of the material. As the shell erodes, the mechanical support to protect the core is lost and this speeds up the degradation of the material. Also, liquids may leave behind substances like salts and other residues in the core, which affects its overall absorption ability. Residues may interact with the polymers in the core and expedite the breakdown of the material’s long-term functionality or may block parts of the core, resulting in diminished absorption ability. The shell might lose its flexibility with time, making it stiffer, brittle and more prone to cracking, minimizing its ability to protect the core. Moreover, the shell’s glassy material becomes more fragile with every absorption and compression cycle, which makes it riskier for it to break or split during use.

**Figure 8:**
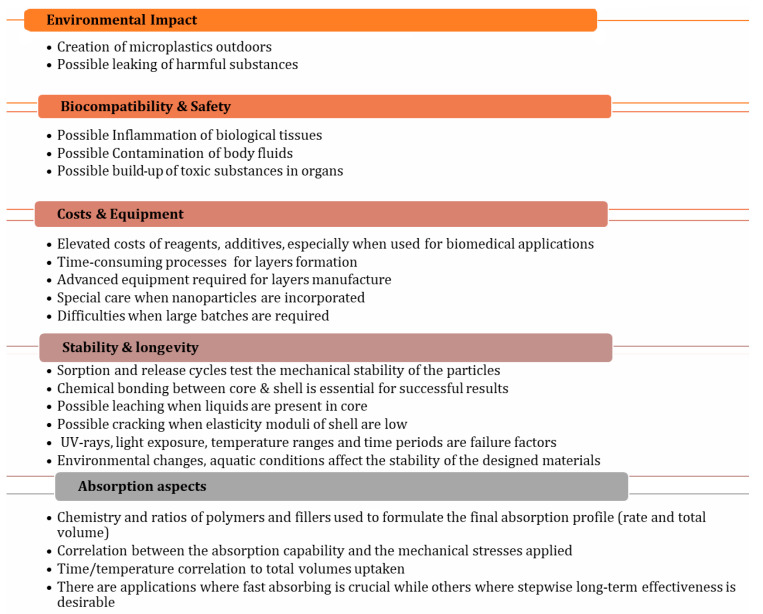
Factors the applications of SACM face in real terms.

Figure 8 consolidates the key factors that the SACM confronts under real-world operational conditions. It highlights the multifaceted challenges impacting system performance, including environmental stressors, material degradation, and operational variability.

### 5.8. Scaling Nanostructures

Core/shell SACMs, often referred to as nanoparticles, consist of multiple nanostructures (<100 nm). It is observed that slight alterations in the conditions of parameters during manufacture processes can produce great heterogeneities in their properties, and difficult synthesis techniques may not be able to be translated to mass production easily. It would be important to advance these processes with a view to making these novel materials have extensive commercial viability.

Core/shell SACMs require advanced and particular technologies to be fabricated, so that the thickness and uniformity of the shell is controlled and formed on a nanoscale [41]. These procedures are difficult and time-consuming. Precision and special equipment are requirements as well, which increases production complexity. Also, consistency and reliability across nanoscale structures are a necessity and require severe quality control processes. The materials that are used in those syntheses are certain types of polymers. The procedures that are most used for achieving nanostructures have more steps and usually require more energy [73,74,75].

Also, it is difficult to achieve nanostructures when scaling up from laboratory to industrial quantities. As production scales up, uniformity in the thickness is hard to maintain, especially in large batches, causing inconsistences in product quality. Moreover, even small changes in the temperature or pressure during production can cause uneven nanostructure formation, that is even harder to maintain in large-scale production [39]. The smaller the particle interactions, the harder the achievement of strong bonds. So, at the nanoscale, interactions are more sensitive to irregularities and imperfections. Due to this peculiar bonding the core and the shell may be detached. So, the use of specialized adhesives or coatings is necessary (as coupling agents) and can increase the complexity of the processes.

Nanostructured core/shell SACMs are often more sensitive to environmental conditions and more prone to degradation when exposed to environmental factors like UV, as stated [71]. So, there is also a need for additional protective layers that can help and that can protect their properties. In situations that require a big surface area, nanoscale materials might not be the most suitable solution, since they do not provide the required absorbency. This happens because some properties of nanostructures are diminished when scaled up.

Ultimately, some nanoscale materials could be life-threatening to human beings when in direct contact with the consumer. All in all, disposing of nanomaterials is often complicated as nanoparticles, which tend to persist in the environment, require specialized disposal processes [58]. When dealing with nanomaterials, the application of more regulations or laws may be necessary. This might also complicate the entire process at other levels.

## 6. Future Perspectives

**Figure 9:**
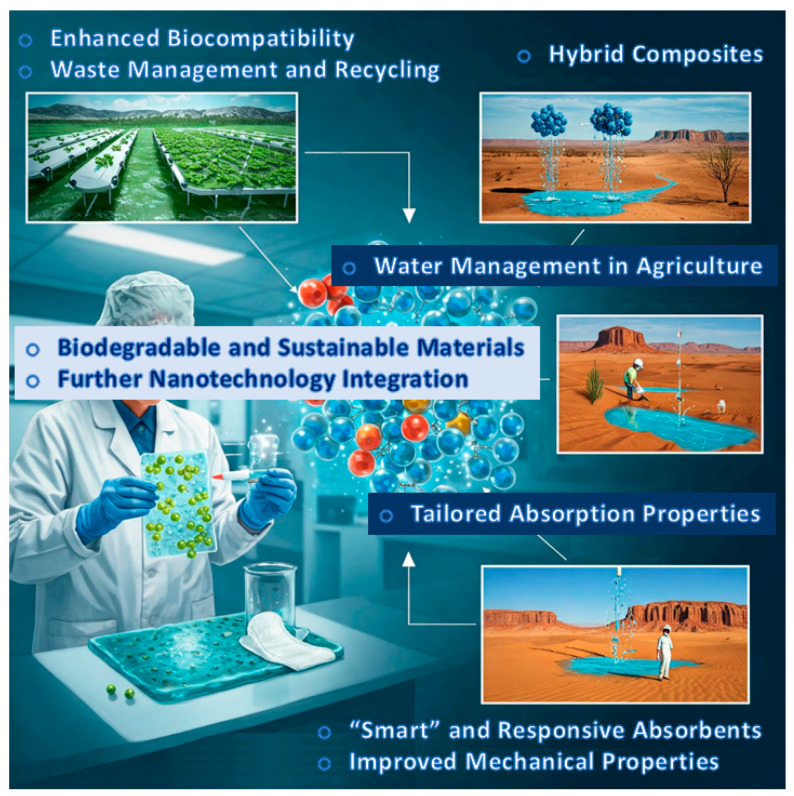
The classification of SACM future perspectives.

Highlighting the future directions currently being pursued by the scientific community in the field of SACMs underscores their significant potential for further advancement. Ongoing efforts are directed toward improving selectivity, stability and scalability to meet industrial demands. The adoption of advanced computational modeling and machine learning is anticipated to expedite catalyst optimization. Additionally, sustainable approaches, including greener synthesis methods and the use of earth-abundant metals, are becoming increasingly prominent (Figure 9).

### 6.1. Towards Biodegradable and Sustainable Materials

Future directions for core/shell SACMs focus on developing biodegradable and sustainable options, such as utilizing bio-based polymers (or bio-sourced polymers or biopolymers) and natural additives to enhance environmental compatibility. Innovations seek to improve biodegradation rates while maintaining absorption efficiency [43]. To begin with, the development of biodegradable polymers for shell material, the outer layer, is essential. Biodegradable polymers can take the place of traditional synthetic polymers to limit long-term damage [76]. Sustainable polymers such as polyhydroxyalkanoates, which are natural polymers, are an excellent “greener” alternative. By modifying biodegradation rates to manage shell life in various aquatic environments, some catalysts could work properly and easily degrade through post-treatment. Thus, this issue makes a great research topic. Plus, an eco-friendly method of synthesis can reduce the use of harmful solvents [9]. Incorporation of aqueous or biomass-derived solvents and “green” agents can lead to this. One way to promote energy-efficient synthesis is by using microwave or ultrasonication techniques, which are effective with less energy consumption [41]. All that will help a lot to reduce the environmental impact as well as energy costs [49].

To enhance the sustainability of these materials, incorporating renewable and natural bio-sourced fillers rather than mineral ones can make the difference [43]. Materials like lignin and bamboo fibers in the shell can improve biodegradability and functionality. Improving the bonding with biodegradable polymers can boost both the durability and retention, thus research like this is a very interesting topic in the field of polymer chemistry and technology [43]. These fillers can create SACMs that are less expensive and have high performance, minimizing the environmental impact [77]. The degradation can also be controlled by mechanisms that can modify their lifecycle, suitable for certain milieu [9]. The use of specific polymers is very important, like pH-sensitive or UV-sensitive polymers that can degrade according to environmental triggers, making sure that they will break down after their effective lifespan [68]. Hydrolysable cross-linkers can also degrade under specific conditions like temperature or moisture change. Achieving nanoscale structures that break under specific conditions is also a topic of discussion and research in the field of nanotechnology.

Techniques like cross-linking are toxic and the exploration of novel “green” and non-toxic techniques is essential [78]. For starters, replacing standard cross-linkers with something more eco-friendly is a great way to reduce toxicity and environmental impact. Studying the effectiveness of natural cross-linkers and comparing them to conventional ones to see if they can have the same absorbency and durability is a topic of investigation as well. Natural cross-linkers can be used, adding stability and strength without harmful chemicals [79]. Enzymatic cross-linking can create bonds in the polymer chain in an eco-friendly process. The techniques are not the only aspects that can be green to reduce the environmental impact [80]. The polymers for the core and the shell can be plant-based and sustainable as well. Research focuses on optimizing polymer extraction and processing methods to increase the scalability because they offer a sustainable alterative for single-use products [81].

### 6.2. Further Nanotechnology Integration

Core/shell SACMs may enhance mechanical and absorption properties using nanoscale modifications, and it is given that integration of nanotechnology is the future of these materials. This refers to the use of nanoparticles for better stability and special performance characteristics. Nanotechnology can enhance the effectiveness of SACMs in different domains of manufacture and industry [82].

Firstly, integration of technology can develop so-called “smart”, responsive core/shell structures by creating SACMs that respond to environmental triggers, like pH and temperature [83]. This can adjust their absorption capacity or even release stored contents on demand [84]. There are thermo-responsive polymers (crucial the T_g_, T_m_ characteristics) that can absorb or release moisture, based on temperature changes [24]. Moreover, there are also pH-responsive materials that can expand or shrink when the pH changes [85]. To achieve high responsiveness and specificity to target molecules, the use of nanostructures and molecularly imprinted polymers is needed. Improving the mechanical resilience of SACMs can make them suitable for reusable applications by enabling multiple absorption–release cycles [9]. To retain the structure after absorbing and releasing liquids, cross-linking agents can be used to strengthen the shell [86]. Incorporated materials like silica or nanoclay can also enhance strength without compromising the absorbency. Developing polymers with adjustable flexibility and strength is a great research topic [9].

Special performance characteristics like antimicrobial and antifouling properties can also be incorporated by developing materials with integrated features, like the ones that are mentioned above [43]. This can be performed by inserting nanoparticles that can reduce bacterial growth within absorbent materials, like silver NPs [87] or ZnO nanostructures. Nanoparticle coatings can be implemented as well, and will have the same effect, without leaching toxic substances. Also, factors like moisture levels, pH and temperature can be monitored by using nanoscale sensors in both the shell and the core, that can detect environmental changes immediately [83]. Color-changing particles (a totally different chemistry) can be incorporated as well within the shell and can respond to moisture or pH variations. These NPs in sensors can be used for non-invasive, real-time monitoring.

Incorporating controlled release mechanisms by SACMs that can absorb and gradually release water or nutrients (or generally active ingredients) over time is improving their efficiency [88] in applications like agriculture. This can be performed by encapsulation of the substance that is going to be used in the core, and then it will be released accordingly, based on the environmental stimuli [83]. Techniques like spray drying are important in massive production, with consistent mass and morphology properties, and adapting synthesis to continuous flow reactors could increase production rates.

Through nanostructuring and surface engineering, swelling control and absorption capacity can be maximized, thus enhancing the surface area and interaction with absorbing materials [40,82]. By using porous shells, the speed and efficiency can be elevated [12]. Water retention can be improved by using functionalized NPs like silica within the shell. Experts in polymer chemistry are adjusting polymer chain lengths and cross-linking density that can further regulate the swelling ratio and absorption capacity [79,86]. Yet, no matter the case, over-swelling must not occur to maintain the integrity of the structure.

### 6.3. Hybrid Composites

Hybrid composites seem to have a great future in core/shell SACMs where properties are enhanced by a combination of materials. This method increases strength and absorption and can be used for different tasks like delivering drugs. Optimizing interactions of the material can increase the stability further [89].

Mechanical strength, thermal stability and water retention are properties that can be enhanced by synthesizing core/shell structures with inorganic NPs, like SiO_2_, TiO_2_ or GO, into the shell layer. SiO_2_, for instance, can improve the stability of the structure and make the materials more durable and, thus, applicable where controlled absorbency is needed [9]. Graphene oxide can boost mechanical strength and responsiveness to environmental stimuli. Optimizing nanoparticle size and surface modification for even dispersion and strong bonding within the polymer matrix is still at a research level [90]. Moreover, bio-based fillers like cellulose nanofibers (CNFs), chitin or lignin can be incorporated within the core or the shell to improve absorbency and biodegradation [91]. To create hybrid SACMs that combine high absorbency along with eco-friendly disposal, they are blended with synthetic polymers, but the procedure is still under investigation [54].

In addition, multilayer core/shell systems can enhance functionality, which certainly needs clarification according to the specific application [46]. Producing core/shell structures with different materials in each layer can be performed in many ways. These structures can enhance properties such as absorbency, controlled release, mechanical support and so on [84]. The absorbent part inside can be made of SAP or hydrogels. The core layer can maximize water uptake and retention. The outer reinforcement layer can be created by durable polymers or nanocomposites to protect them from mechanical stress [84]. A mid-layer made of pH-responsive or temperature-sensitive materials can also be incorporated to release the absorbed substance on-target [92]. Metal–organic frameworks can be used in absorption and selectivity to increase the selectivity of certain ions or molecules. Nanotechnology emphasizes using hydridic structures with uniformly distributed metal–organic frameworks in a polymeric matrix to offer stability and integrity [89].

Different polymers can be combined within the core and the shell to achieve adjustable swelling, absorbency and mechanical properties [92]. Hybrid material incorporates two different phases of the same function that work together but are not necessarily bonded chemically. For instance, two types of filler may create a hybrid filler phase, while a polymer and a filler create a composite material. Misconceptions are often made because of the great development of materials science, so literature searches are inadequate. Blending (which refers to the actual polymeric macrochain and not the subsequent cross-linking) can be between hydrophobic and hydrophilic polymers, allowing controlled water uptake and release, modifying the material for hydrophilic applications [93]. Also, interpenetrating polymer networks can achieve high absorbency along with structural strength. However, it is hard to ensure that the polymer blends remain compatible and stable under every environmental condition. Thermal and chemical stability can be further improved by hybrid nanocomposites [84]. Nanocomposites including SiO_2_ and BN can improve heat resistance and provide isolation, while ZrO_2_ particles can boost chemical resistance. Nevertheless, thermal and chemical resistance may affect the material’s absorbency properties [36].

Finally, developing hybrid SACMs with hydrophobic shells can promote their ability to selectively absorb oils and eject water, a property useful in environmental cleanup and oil-spill management [89], allowing the SACM to repel water, whereas the core can be porous to optimize high-oil absorbency while the shell can selectively reject water [11]. These hybrid SACMs can improve the selective oil absorption capabilities and could provide a sustainable solution to oil–water separation for environmental cleanup.

### 6.4. “Smart” and Responsive Absorbents

The future development of so-called “smart” absorbents that respond to the environment is reported for core/shell SACMs. “Smart” materials can be designed to change their properties in response to changes in conditions like pH or temperature and can thus also control absorption and release. This ability improves their performance in applications such as drug delivery and moisture management, making them more versatile and effective [36,92].

Stimuli-responsive polymers can be used for target swelling and deswelling. Their behavior can change based on pH, temperature and moisture [83]. Thermo-responsive polymers can be used in the shell and can either swell or contract, based on temperature changes. Plus, pH-sensitive polymers can be placed in the core or in the shell to enable the SACM to expand or release contents at a specific pH. SACMs can inspire applications that require adaptive behavior like agriculture. To monitor in real-time environmental conditions such as pH or temperature, sensors can be implanted within the SACMs. Once a threshold is reached, a response is triggered and these materials can monitor the conditions. These sensors can either be activated by moisture and can trigger visual or electronic signals once saturation is reached or be colorimetric and use color-changing substances to indicate moisture levels or pH changes visually. It is truly like incorporating a device which emits signals with the particles manufactured. Nanotechnology mainly focuses on implementing nanosensors to detect environmental changes with high sensitivity and minimal power requirements [36].

Additionally, there has been the development of SACMs with controlled release mechanisms that respond to environmental stimuli, promoting the gradual release of absorbed substances like water [94]. There are specialized core/shell designs with semi-permeable shells, that enable controlled diffusion of nutrients and biodegradable pH-sensitive coatings. pH-sensitive shells can further degrade or become permeable at specific pH levels. Still, achieving precise control over release rates is very difficult.

“Tunable” absorption can become possible by electro-responsive components as well. Developing SACMs that can regulate their absorption properties in response to an electric field can allow precise control over absorption [95]. This is possible by utilizing conductive polymers or incorporating carbon nanotubes in the shell to create SACMs that are electro-responsive, thus when subjected to an electric field their whole structure will change [82]. Optimizing the stability and conductivity of these materials is still under examination to ensure reliable control over absorption and desorption cycles. Moreover, their suitability for complex and variable environments can be increased by creating SACMs that respond to multiple environmental stimuli as before, but this time simultaneously. Hydrogels can be produced by combining thermo-responsive, pH-sensitive and photo-responsive components, allowing them to adapt to different conditions [96]. Also, layered shells that react to different stimuli can be utilized, providing a sequence of responses depending on the combination of conditions. Nevertheless, managing interactions between responsive components to prevent undesirable mixed responses is a very complicated thing that is still under examination.

Lastly, self-regulating absorption and release depending on environmental conditions can be performed by producing SACMs inspired by natural systems, like plant root systems. This procedure can be carried out by creating materials that ideally absorb water based on water needs, similar to how plant roots adapt to moisture availability, thus mimicking plant hydrotropism, or by designing polymers that expand in response to saturation levels, allowing SACMs to maintain optimal content autonomously. So, these “bio-inspired”, “bio-mimicking” SACMs can adapt to changing conditions, which is ideal for irrigation systems or agriculture solutions [97].

### 6.5. Improved Mechanical Properties

Enhancing mechanical properties is a key direction for core/shell SACM, as is the case for all novel materials that can be made for advanced applications. By optimizing core/shell structures and material interactions, these composites can better withstand pressure and environmental stress, expanding their use in demanding applications.

The first thing to improve mechanical properties is to incorporate high-strength nanomaterials, like GO or carbon nanotubes (CNTs) for reinforcement. If these materials are integrated into the shell, the mechanical strength of the SACMs will increase without compromising their absorbency [98,99]. Both GO and CNTs offer high tensile strength and elasticity, thus the material can withstand the mechanical stress during swelling. SiO_2_ NPs can also be used due to their stiffness and resistance to deformation, moreover they do not cause harm to any living organism or the environment [9]. These SACMs have superior strength and could be ideal for applications that require high resilience in mechanical stress, even though it is hard to achieve uniform dispersion of nanomaterials within the polymeric matrix, if added alone [84].

Another way to improve mechanics, in the absence of reinforcing agents, is by cross-linking polymer chemistry. Advanced cross-linking techniques may be used to increase the elasticity and structural stability of SACMs [86]. This prevents integrity loss during repeated swelling and drying. For example, it could be due to reversible covalent bonds in the shell that enable “self-healing” under harsh conditions or due to ionic interactions, that could lead to strong flexible network formation for enhanced durability. All that needs to happen without compromising the absorbency or the response time. That is hard but, eventually, these SACMs will have applications where high elasticity is needed in the sense of recovery of the energy applied.

SACMs with multilayered core/shell architectures can be designed for distribution of the mechanical load to the core, improving shock absorption and enhancing resilience during compression and expansion [77]. In order to achieve that, the inner layer of the shell has to be softer, and the outer layer of the shell has to be stiffer, thus mechanical stress will be evenly distributed, reducing strain [67]. Strain over the limits results in cracking, when the particles cannot go from their deformation back to the initial state. Also, hybrid core/shell structures can be designed by combining materials with varying elasticity in each layer, improving the material’s ability to absorb and release fluids [84]. Furthermore, “self-healing” polymers can also be incorporated to allow them to recover from minor tears or structural damage, enhancing longevity and sustainability. Encapsulating healing agents within the shell that release upon damage is a great way of initiating repair. Those materials can be used in applications that require extended lifespan and minimal maintenance [100,101].

The thickness of the shell and the density of the core can also be designed to accomplish a balance between mechanical strength and absorption efficiency [12]. A thicker shell layer can prevent rupture during swelling and increase durability, while densely packed core particles or fibers can make the structure more stable, supporting the shell during fluid absorption and release [90]. Additionally, utilizing blend polymers in both core and shell to combine the benefits of toughness and flexibility can create SACMs that are durable and adjustable. Tough and flexible polymers can be combined, improving their toughness without sacrificing flexibility [92]. Also, composites of thermoplastic and elastomer parts can give SACMs a perfect balance between elasticity and durability, allowing better resistance to mechanical stress.

Not only mechanical but also thermal stability can be enhanced by hybrid organic–inorganic composites. Developing SACMs with these composites can leverage both types of materials for superior mechanical and thermal stability [84]. Nanoparticles like SiO_2_ and clay can provide rigidity and thermal resistance, helping SACMs withstand high-pressure or high-temperature environments, while CaCO_3_ or Al_2_O_3_ particles can be incorporated to improve compressive strength and stability. The difficult part that is still under research is the incorporation of inorganic materials into the polymeric matrixes, without compromising absorbency or causing brittleness.

Mechanical performance and resilience can also be boosted by implementing supermolecular chemistry into SACMs to produce materials with high-strength, reversible bonding. Hydrogen bonding or π–π stacking can create strong but reversible bonds and this can help the material withstand mechanical stress. Also, host–guest interactions in the core or in the shell can allow reversible bonding and this can give the material flexibility under stress, so its resilience is improved.

### 6.6. Enhanced Biocompatibility

Improving biocompatibility in core/shell SACMs is essential for safety reasons. Selecting non-toxic reagents and solvents and reducing harmful additives enhances their compatibility with biological tissues, minimizing immune responses or inflammation that could compromise their effectiveness or safety [47,94]. Enhanced biocompatibility not only protects surrounding tissues but also ensures the material performs as intended over time, without triggering adverse biological reactions [92]. This compatibility is crucial to support wider use in sensitive applications like drug delivery, where the material must safely interact with biological fluids; in wound care, where it must promote healing without irritation; and in implants, where long-term tissue integration and stability are vital for patient health [88].

For starters, biocompatible polymers can be utilized for both the core and shell to prevent adverse reactions [54]. The best choice is natural polymers, such as alginate or chitosan, because they are well-tolerated in biological environments, making them perfect for medical applications [43]. Moreover, synthetic biocompatible polymers like poly(vinyl alcohol) are useful and do not provoke immune responses as well. Incorporation of such polymers in SACMs will expand their use in applications like healthcare, reducing the risk of irritation, toxicity or even inflammatory responses. To achieve the latter, embedding anti-inflammatory agents within the SACM structure will have the same effect. This will minimize immune response upon contact with biological tissues. Natural anti-inflammatory compounds like aloe vera can be encapsulated within the SACM, allowing controlled release to reduce the inflammation, whereas synthetic anti-inflammatory agents like hydrogels can provoke localized anti-inflammatory effects [92]. Nowadays, developing slow release formulations that release anti-inflammatory agents over time to improve patient comfort and healing rates is a focus in the field of polymer chemistry.

Mimicking biological tissue compatibility can improve adhesion and minimize irritation. This can happen by engineering the surface chemistry and morphology of SACMs by some techniques. Zwitterionic polymers can coat SACMs and can reduce protein adsorption, preventing immune recognition and adhesion [78]. A hydrophilic surface can reduce friction, making SACMs more suitable for sensitive applications, due to the fact that they are less irritating. Nanotechnology also deals with research concerning the control of roughness and chemical groups that interact with biological tissue through nanostructuring the surface at the molecular level.

To prevent infections, antimicrobial agents can be utilized by integrating them into the SACMs [43]. Silver nanoparticles and ZnO are nanomaterials that have effective antimicrobial properties. Natural antimicrobial agents like chitosan are also biocompatible and can reduce the risk of inflection without contributing to antimicrobial resistance [92]. However, balancing antimicrobial efficiency with absorbency can affect material properties. Moreover, it is very important to apply biodegradable materials to ensure that SACMs degrade safely in the body without causing adverse effects [43]. Materials like PCL can be elaborated, because it degrades naturally in the body, while biocompatible polymers like collagen provide a structure that degrades without causing irritation. Applications like temporary implants, surgical dressings or single-use medical items can benefit from such materials.

Safe delivery and release of active ingredients like nutrients can allow controlled therapeutic or regenerative actions [81]. Once again, pH-responsive or temperature-responsive polymers can release active ingredients in response to the body’s specific conditions. Specific pH changes and layer-by-layer coatings use a technology-based approach that can gradually release active compounds for long periods of time. Developing a biocompatible polymer matrix that can hold and release active agents in a controlled way is a novelty of polymer chemistry [84]. These properties can be helpful in fields like regenerative medicine.

To avoid additional adhesives, SACMs with “bioadhesive” properties may be made to attach materials to the tissue surfaces. Mussel-inspired polymers mimic a natural adhesive and can provide strong and biocompatible adhesion, thus they are a great alternative. Gelatin-based adhesives are an alternative as well, due to their bond formation properties with skin and tissue. Nevertheless, developing such SACMs that can adhere only under specific conditions, to biological surfaces and in biological conditions in particular, is still under investigation [102].

As illustrated above, SACMs can cause allergies, so the use of non-irritating, hypoallergenic materials will minimize these allergic reactions. Thus, they are suitable for people with sensitive skin. Silicone-based chemical substances, used in skin creams and medical plasters, or generally non-toxic natural polymers like agarose are hypo-allergenic and do not cause allergic reactions [93]. Chemistry research focuses on creating these materials and making them free from leaching chemicals that cause irritation.

### 6.7. Waste Management and Recycling

The focus of development in waste management and recycling of SACMs must be on environmentally friendly, recyclable designs to reduce waste and improve reusability [9]. With the use of biodegradable material like plant-based polymers, SACMs can degrade naturally. Moreover, designs that facilitate material separation make recycling easy; the core and shell layers can be hypothetically separated after use, allowing their components to be composted or recycled. Selective degradation techniques where bio-based additives respond to environmental effects such as moisture and pH to control degradation in soil or water are also explored. These methods help various sectors—including agriculture, aquaculture and healthcare—dispose of waste sustainably and minimize landfill use [103].

The most important thing is to develop SACMs that have minimal environmental impact by prioritizing recyclable and compostable components that relieve waste management [40]. Using nanotechnology, hybrid materials that can maintain performance while being reused can be a great target for research. Also, incorporating biodegradable polymers, like plant-based or bio-derived polymers, can reduce long-term waste and minimize landfill accumulation. These polymers can once again be PLA or polyhydroxyalkanoates due to their ability to degrade naturally. Thus, SACMs will leave little to non-assistant environmental residues, making them suitable for disposable applications [104].

Moreover, SACMs that can be easily separate into individual components are good to be created. These components may be directed to different recycling or disposal streams and this approach expedites the proper recycling of multimaterial SACMs [76]. One potential research approach would be to produce core and shell layers that can be detached through nanotechniques, that will help separation without compromising the structure during their use. Nanotechnology focuses on utilizing techniques to create non-permanent bonds between the layers that detach when they are in a specific environment. Polymer coatings that respond to pH changes or heat can be included.

Using the same mindset, degradation via environmental triggers can happen, enabling SACMs to degrade gradually and selectively, without premature breakdown during their use [99]. Environmental triggers can be moisture, pH or temperature change. pH-responsive hydrogels or thermo-responsive biopolymers can be incorporated into the SACM structure to maintain stability in natural pH but once the material is in an acidic or basic environment they will break. SACMs with properties of selective degradation can be useful in applications like agriculture [101].

Properties like compostability can be enhanced by incorporating bio-based additives that are compostable and can improve the degradation of the SACMs, assisting rapid decomposition when used. Exploring bio-based additives, minerals or organic compounds can support faster degradation. These materials can either be natural fibers like flax or CaCO_3_ and can be chosen to degrade quickly, contributing to soil health. Materials with such properties can be used in applications where post-use disposal includes soil or compost environments and can not only add to the environmental value but also enhance the quality of the compost.

Nanoscale engineering can be used to enhance the absorbency and efficiency of SACMs, allowing lower material use and at the same time maintaining high performance, reducing overall material waste [69]. Thinner and more absorbent SACM layers can be created to reduce raw materials but not lower their efficiency. Nanoparticles’ coatings and nanoscale structures can enable the use of less overall material, due to the creation of ultraabsorbent SACMs. Thinner material layers with optimized absorption properties that could reduce the cost of production and decrease waste is one more topic of polymer chemistry. All of that can support sustainable production and reduce environmental burden.

### 6.8. Water Management in Agriculture

Developments in these composite materials enhance water retention and controlled release, supporting efficient agricultural water management. Improved absorption properties help sustain soil moisture, reduce water consumption and increase crop resilience in dry conditions by gradually releasing moisture to plants [105]. This minimizes irrigation frequency, supports steady root hydration and helps young plants thrive, despite irregular rainfall or high temperatures. By conserving water, these materials reduce farming costs, lessen strain on water sources and, all in all, promote sustainable agricultural practices critical for food security in the face of climate variability.

Water use can be optimized by using SACMs that gradually release absorbed water to plants, supporting crops during dry periods. Some polymers can release water based on the soil’s pH or osmotic gradients (since they are overall heterogenic systems), reducing water waste. Such materials are pH-responsive polymers or osmotic-controlled materials [105]. Also, layered core/shell structures can slow down the water release, thus the deeper hydration of soil continues for a long period of time. The difficult part is to balance the water release rate with the material’s water retention capacity to avoid quick drying [106]. Moreover, soil nutrients like N or K can be included in the SACMs. This can enable SACM to release both water and nutrients when this is required and may be realized by encapsulating fertilizers within the core, that will be released along with water or by nutrient-sensitive shell structures in the form of coatings that will degrade according to the environmental conditions and release specific nutrients. This method requires a precise release of nutrients to avoid nutrient turn-off [107].

Soil pollution can cause long-term residue build-up. One way to solve this problem is by utilizing bio-based and biodegradable polymers, that are mentioned above [108]. Biodegradable SACMs can improve soil quality and sustainability, making them perfect for environmentally conscious farming that can reduce plastic waste [109]. Furthermore, farmers can monitor the soil water content in real time and adjust irrigation as needed. This can become possible by integrating moisture-sensing elements within SACMs, like polymers that change color based on water levels or by nanosensors that communicate moisture levels to a central system. However, it is not easy to ensure sensor stability and data accuracy in different soil conditions.

Enhancing water absorption and swelling capacity can support weather resilience. By boosting such properties, SACMs can hold larger water volumes, making sure that plants have access to water in prolonged droughts [110]. The ways of enhancing absorption rates and water retention are analyzed above. Superabsorbent hydrogels with high swelling capacity can retain more water for longer, buffering the crops against harsh weather [111]. Also, nanoscale pores can increase the surface area and improve water uptake, allowing fast response to heavy rainfall. Nevertheless, it is hard to avoid excessive swelling that can disturb soil structure or root development, when it comes to ground watering and outdoors conditions [112,113].

Soil’s health can also be preserved by developing SACMs with antimicrobial properties to prevent the growth of harmful microbes in wet soil conditions [114]. Natural antimicrobials like chitosan can be incorporated into SACMs and can prevent microbial growth without harming beneficial soil organisms. Bio-based NPs like ZnO can also be utilized, due to their strong antimicrobial effects. Antimicrobial SACMs can reduce crop losses due to soil-borne diseases, supporting healthy plant growth [115,116].

Not every place on Earth has the same climate and soil conditions, so in the creation of specialized SACMs with tailored properties like absorbency or biodegradability, specific ingredients to fit the unique conditions of different regions are of high importance [117]. For arid regions, drought-resilient SACMs can be utilized to focus on maximum water retention and slow release mechanisms, while for tropical climates SACMs with high absorption and quick release to accommodate heavy rainfall can be used. The modification of the polymer composition and shell properties to suit specific pH, salinity and climatic conditions is a great focus on the field of chemistry [118].

### 6.9. Tailored Absorption Properties

Tailoring absorption in core/shell SACMs involves adjusting core/shell structures to control water uptake, retention and release rates. Customizable properties, like pH-responsiveness or temperature sensitivity, enhance SACMs for precise applications in agriculture, medicine and water management. These adjustments allow for targeted, efficient moisture delivery, whether in sustained crop hydration or gradual release in wound care, optimizing both performance and sustainability [119].

As mentioned before, SACMs with adjustable absorption rates in response to environmental stimuli can be created. Incorporation of temperature-responsive materials like hydrogels can increase absorption capacity as temperature rises [84]. Also, pH-sensitive core materials can adjust absorption rates based on soil or water pH. Manufacturing shell materials that can absorb large quantities of water with no excess swelling can preserve material structure and integrity [120]. Cross-linked nanofiber networks can prevent expansion by maintaining the shape whereas elastic polymeric coatings might expand a little but will overall prevent excessive swelling [40]. These materials with controlled swelling properties could be useful in applications like agriculture, as previously analyzed [9]. However, too much stiffness has to be avoided, especially in applications in which flexibility is required.

Furthermore, SACMs can be designed in a certain way to be able to absorb water quickly for immediate hydration requirements like in case of a fire. To achieve this, porous matrixes have to be designed, that will promote fast water uptake, making them suitable for emergencies [11], or hydrophilic NPs like SiO_2_ incorporated in the core to increase the water absorption rate.

To meet specific application requirements some absorption capacities must be adjusted [120]. These tailored SACMs can have various properties that can be used in a variety of fields, such absorbent SACMs for arid agriculture as mentioned above or for low-absorbance options for controlled hydration [9]. This is performed by altering cross-link density in the core, so the absorbency capacity can be controlled by the handler, or by combining polymers with different absorption rates to create a range of absorption.

Nanoporous shells can upgrade water retention and provide progressive water release, so it is crucial that SACMs with such shells are designed [11]. This will enhance the material’s efficiency over time and can be performed by either using nanoporous coatings that can trap water within the core and can constrain the release to a slow rate or by utilizing microchannels allowing the water to seep out little by little. Despite everything, unreasonable complexity has to be avoided, and compatibility between core and shell materials has to be guaranteed.

On top of that, to absorb specific types of liquids like oils in agriculture, environmental remediation and industrial applications, shell surfaces can be optimized to pull specific liquids based on polarity or ionic concentration [9,83]. SACMs with selective absorbency can serve niche applications like nutrient delivery. Absorption based on temperature change can be achieved as well [103].

Creating SACMs that transform their absorption properties based on a change in temperature can be accomplished by utilizing thermo-sensitive hydrogel layers to expand or contract in response to temperature or by incorporating phase-change materials that either absorb or release water at a specific temperature. Such SACMs would provide seasonally adaptive moisture management, that is ideal for agricultural purposes [121].

## 7. Conclusions

The literature search conducted presents valuable findings regarding the components, the applications and the parameters affecting the functions of superabsorbent polymers and composite materials. It was found that the fields of environmental technology, medical applications and packaging industry enjoy incorporating not only hydrophilic materials but materials that can retain much water in their structures. The cycle of absorption and releasing of moisture depends on parameters like pH, temperature, time of activity, active ingredients and salts, light exposure, presence of inorganic fillers and microstructure. Yet, adjustments are possible regarding the exact applications, revealing the wealth of adaptations these materials may allow.

The second part of this review covers the challenges these materials face in terms of longevity and mechanical endurance which need amelioration for certain applications, while high cost and environmental pollution issues are evident, as well. Furthermore, the research trends that are discussed in recent literature are summarized, including biodegradability issues, tailored qualities and water management when in great quantities.

Generally, superabsorbent polymers and composite materials are considered valuable advanced materials and state-of-the-art technology for multiple demanding applications and will be proved to ally to numerous natural and artificial procedures where water absorption is considered essential.

## Data Availability

Data sharing is not applicable to this article as no new data were created or analyzed in this study.

## Abbreviations

The following abbreviations are used in this manuscript:

BET	Brunauer—Emmett—Teller
CNFs	Cellulose nanofibers
CNTs	Carbon nanotubes
CTAB	Cetyltrimethylammonium bromide
DLS	Dynamic light scattering
DSC	Differential scanning calorimetry
EDS	Energy dispersive X-ray elemental analysis
EIS	Electrochemical impedance spectroscopy
FT-IR	Fourier transform infrared
GO	Graphene oxide
MN	Microneedle
MWCNTs	Multiwalled carbon nanotubes
NFs	Nanoflowers
NMR	Nuclear magnetic Resonance spectroscopy
NPs	Nanoparticles
PAA	Poly(acrylic acid)
PCL	Poly(*ε*-caprolactone)
PDA	Polydopamine
PES	Polyethersulfone
PVA	Polyvinyl alcohol
SACM	Superabsorbent composite material
SEM	Scanning electron microscopy
TEM	Transmission electron microscopy
TEOS	Tetraethoxysilane
TGA	Thermogravimetric analysis
UV-Vis	Ultraviolet–visible
VSM	Vibrating sample magnetometry
XPS	X-Ray photoelectron spectroscopy
XRD	X-Ray diffraction
